# Dynamics of respiratory viruses other than SARS‐CoV‐2 during the COVID‐19 pandemic in Madrid, Spain

**DOI:** 10.1111/irv.13199

**Published:** 2023-09-26

**Authors:** Patricia Brañas, Irene Muñoz‐Gallego, Elena Espartosa, Noelia Moral, Guadalupe Abellán, Lola Folgueira

**Affiliations:** ^1^ Microbiology Department Hospital Universitario 12 de Octubre Madrid Spain; ^2^ Biomedical Research Institute imas12 Hospital Universitario 12 de Octubre Madrid Spain; ^3^ Department of Medicine, School of Medicine Complutense University Madrid Spain

**Keywords:** epidemiology, influenza, respiratory syncytial virus, respiratory viruses, SARS‐CoV‐2

## Abstract

The COVID‐19 pandemic and the implemented control measures have impacted the circulation of respiratory‐transmitted pathogens. In this report, we present data from a retrospective study that included 17,883 specimens conducted between 2018 and 2022 in our facility, describing the dynamics of circulation of the main respiratory viruses. We observed a significant decrease in all viral detections (other than SARS‐CoV‐2) starting from March 2020. However, rhinovirus maintained comparable levels to the pre‐pandemic period. Additionally, influenza viruses were not detected during the 2020–2021 season, and respiratory syncytial virus (RSV) exhibited a shift in its seasonality, with an epidemic peak occurring in the summer of 2021.

## 1 INTRODUCTION

Respiratory infections are a leading cause of morbidity and mortality worldwide, and viruses account for a significant proportion of these infections. Respiratory viruses typically show stable circulation and seasonality.^1^, ^2^ However, the emergence of the COVID‐19 pandemic in early 2020 has had a significant impact on the circulation of other respiratory viruses.

This study aims to investigate the dynamics of the different respiratory viruses other than SARS‐CoV‐2 from March 2020 to August 2022 in Madrid, a region with a high incidence of SARS‐CoV‐2 and strong implemented control measures.

## 2 MATERIALS AND METHODS

This retrospective study was conducted at Hospital Universitario 12 de Octubre, a tertiary care facility in Madrid, Spain, whose virology laboratory is a reference regional laboratory for Flu, RSV, and SARS‐CoV‐2 surveillance in primary care settings (Acute Respiratory Infection Surveillance Network). All patients attended at the emergency room (ER), clinical services, or primary care between September 1, 2018, and August 31, 2022, and tested positive for any respiratory virus other than SARS‐CoV‐2 (Flu A, B, RSV, RV, human metapneumovirus [hMPV], adenovirus [AdV]], or parainfluenza virus [PIV] types 1 to 4 were included).

According to the protocols established at our center prior to the pandemic, only samples for Flu or RSV were tested during their respective seasonal circulation periods, guided by the available epidemiological data at each moment. Regarding screening for other respiratory viruses, samples were exclusively accepted from pediatric patients (≤18 years) admitted because of respiratory infections, individuals of any age with immunodeficiency conditions (hematological disorders, cancer, or transplant recipients), or patients of all ages referred from the Primary Care of the Acute Respiratory Infection Surveillance Network.

Nasopharyngeal specimens were processed by multiplex real‐time PCR using the Panther Fusion® assay (Hologic Inc., San Diego, CA, USA). Three diagnostic panels were utilized: (1) Fusion Flu A/B/RSV, (2) Fusion AdV/hMPV/RV, and (3) Fusion Paraflu (1/2/3/4). In cases where a sample tested positive for Flu A virus, subtyping was performed using real‐time PCR with the Allplex™ assay (Seegene, Seoul, South Korea).^3^

For the statistical analysis, quantitative data were presented as the median with interquartile range and qualitative variables were represented using absolute and relative frequencies. Categorical variables were compared using the *χ* ^2^ test, and continuous variables were analyzed using either the Student's *t*‐test or the Mann–Whitney *U* test, as appropriate.

## 3 RESULTS

A total of 17,883 patients were included in the study, among whom 6610 (37.0%) tested positive for a respiratory virus. The specimens underwent testing using one or more diagnostic panels based on clinical and epidemiological criteria. Specifically, 17,653 samples were analyzed using Panel 1, 7869 samples using Panel 2, and 7532 samples using Panel 3.

**FIGURE 1:**
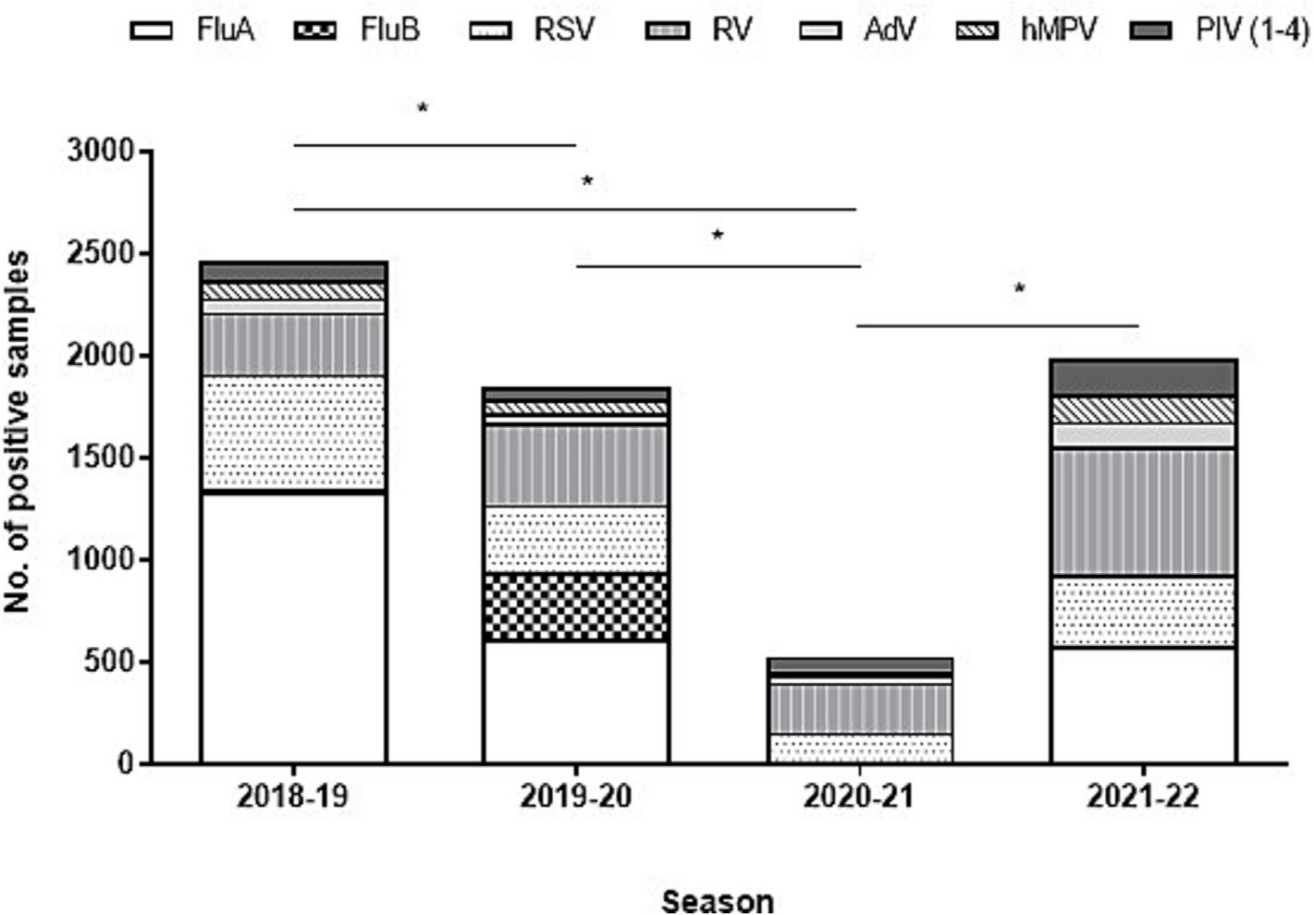
Prevalence of different respiratory viruses by season (2018–2022). *Statistically significant differences (*P* < 0.00001) were found in terms of the decrease of detected viruses between the 2018–2019 season and the 2019–2020 and 2020–2021 seasons, as well as in the increase observed in viral detections in 2021–2022 compared with 2020–2021. Each annual season is shown from September to August. Flu A, influenza A; Flu B, influenza B; RSV, respiratory syncytial virus; RV, rhinovirus; AdV, adenovirus; hMPV, human metapneumovirus; PIV (1–4), parainfluenza virus (1–4).

Globally, the most frequently reported virus was Flu A (*N* = 2538/17,718), followed by RV (*N* = 1575/7594) and RSV (*N* = 1406/17,718) (Figure 1). However, RV had the highest relative frequency among the tested samples, with a positivity rate of 20.7%.

**FIGURE 2:**
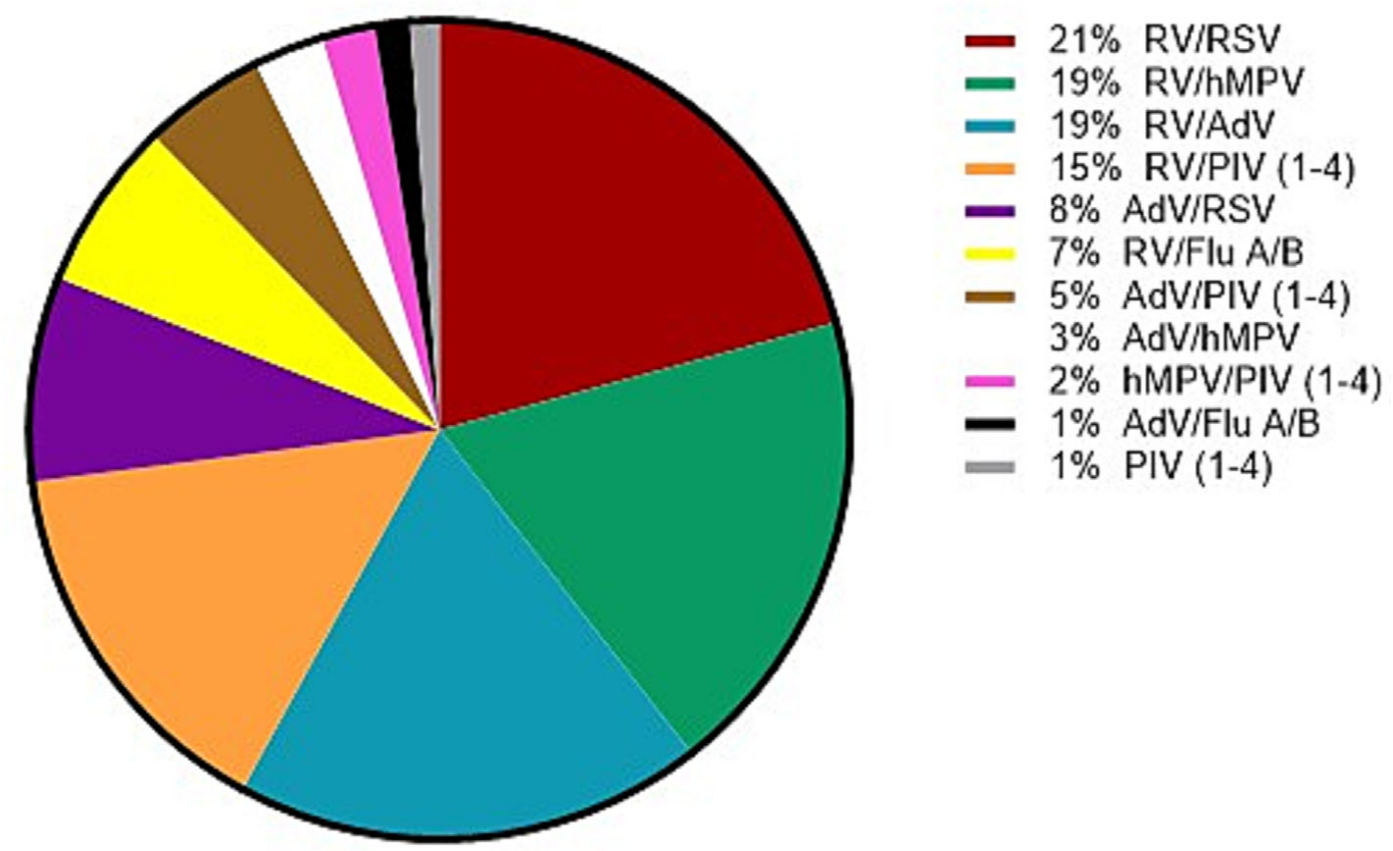
Co‐detections of different respiratory viruses found in the study. Flu A, influenza A; Flu B, influenza B; RSV, respiratory syncytial virus; RV, rhinovirus; AdV, adenovirus; hMPV, human metapneumovirus; PIV (1–4), parainfluenza virus (1–4).

In 433 samples (3.1%), ≥2 viruses were detected simultaneously, and this was more commonly observed in children (*P* < 0.05). RV was involved in 79.7% of these co‐detections and was found with other viruses at various frequencies (Figure 2).

**FIGURE 3:**
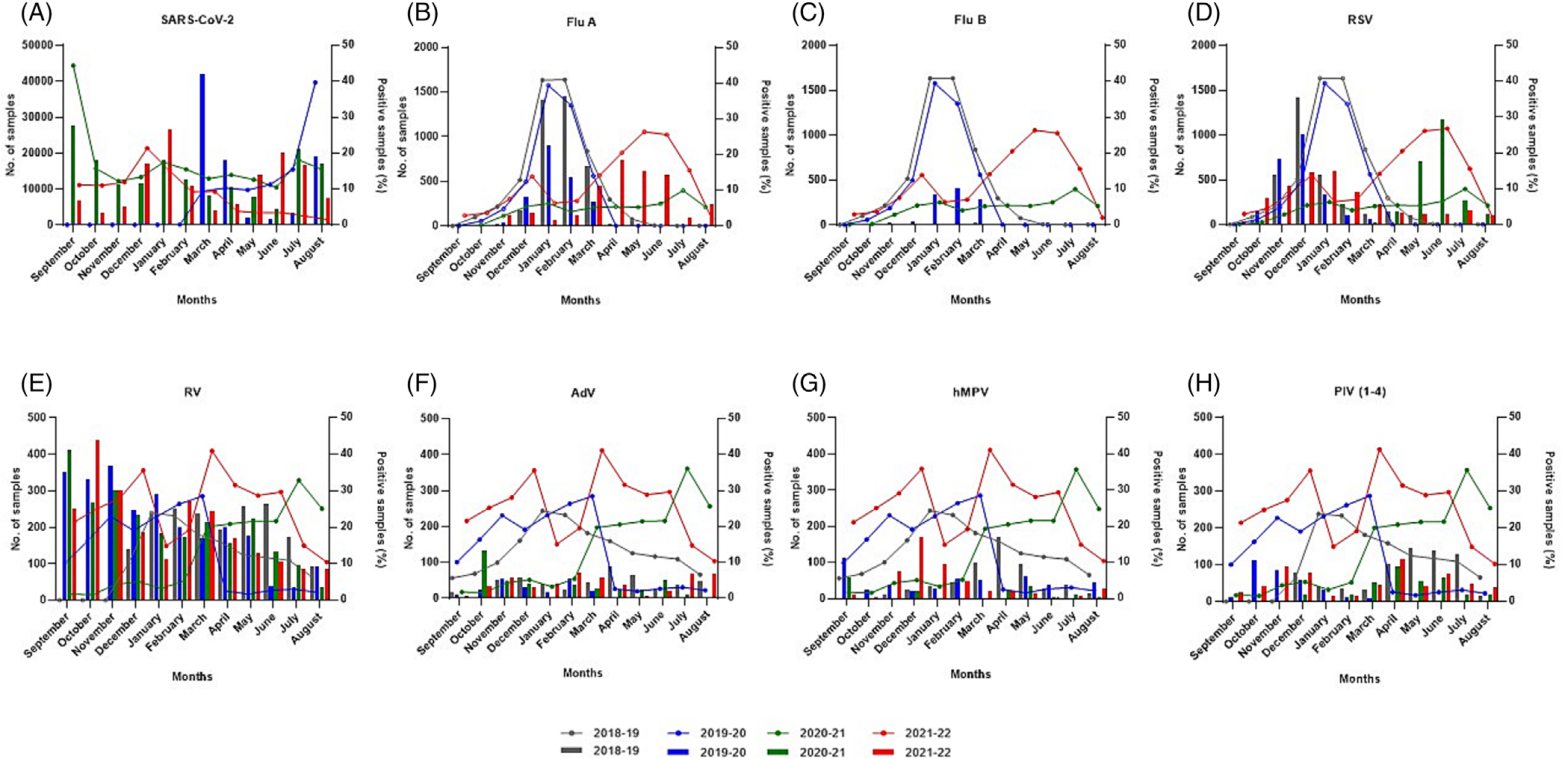
Trend in the number of respiratory viruses detected from September 2018 to August 2022. The bars represent the positive samples (%), and the lines are the total number of samples processed for each of the viruses studied. Each color corresponds to a season: gray (2018–2019), blue (2019–2020), green (2020–2021), and red (2021–2022). Each annual season is shown from September to August. For SARS‐CoV‐2, data are shown since February 2020, when testing for this virus was initiated. No data are available for rhinovirus detection between September and November 2018. Flu A, influenza A; Flu B, influenza B; RSV, respiratory syncytial virus; RV, rhinovirus; AdV, adenovirus; hMPV, human metapneumovirus; PIV (1–4), parainfluenza virus (1–4).

Before the emergence of SARS‐CoV‐2, both influenza viruses and RSV followed a typical seasonal pattern. RSV was predominantly detected from October to February, with a peak incidence in December for pediatric cases and January for adults. In contrast, the circulation of influenza viruses occurred between December and March, with its peak occurrence typically in January and February, varying depending on the season (Figure 3B–D). During the 2020–2021 season, Flu circulation disappeared, but Flu A reappeared in November 2021, reaching a maximum peak detection of 18.3% (150/820) in April 2022 (Figure 3B). Only two Flu B‐positive specimens were identified after the onset of the pandemic. Among the 2538 Flu A strains, 2295 (90.4%) could be typed. The subtypes alternated between seasons, with the majority being A (H3N2) in 2018–2019 (850/1248) and A (H1N1) pdm09 in 2019–20 (472/522). In the 2021–2022 period, A (H3N2) was almost exclusively identified among all typed isolates (520/525). RSV was detected at high levels during the spring and summer months of 2021, with a peak detection rate of 29.4% (77/262) in June 2021, followed by a decrease to 14.8% (38/256) in January 2022 (Figure 3C).

RV was the only virus detected at pre‐pandemic levels in both 2020 and 2021, except for the months of May to August 2020 (Figure 3E). The other viruses were primarily detected from September 2021 onwards (Figure 3F–H).

Flu A was the most prevalent virus among the adult population, detected in 1981/12,737 patients (15.5%), whereas RV was mainly identified in children (909/2971). Among pediatric patients, 50.2% were male, and among adults, 47.8%.

Patient age varied depending on the respiratory virus identified. In children, the median age for RSV was 0.6 years (IQR: 0.2–1.8), significantly lower (*P* < 0.001) than the age observed for other viruses. When comparing the age of children with positive RSV detection before and after the pandemic, a lower median age was observed in the pre‐pandemic group (0.5 years, IQR: 0.1–1.6) compared with the pandemic group (0.9 years, IQR: 0.3–1.9) (P = 0.0002). Among adults, patients with RSV had an older median age of 73.6 years (IQR: 58.1–85.7) compared with other pathogens. Complete data on age distribution and the detected viruses can be found in Table 1.

**TABLE 1:** Comparative analysis of respiratory virus distribution between adults and children: contrasting patients admitted to the hospital or seen at the emergency room with those from primary care in both age groups.

	Adults (*N* = 12,737)					Children (*N* = 5146)				
	Age (years)	All (%)	PC (%)	H/ER (%)	*P*‐value	Age (years)	All (%)	PC (%)	H/ER (%)	*P*‐value
Flu A	61.0 [40.1, 78.7]	1981 (15.5)	346 (12.4)	1635 (16.5)	**<0.001**	4.2 [1.6, 9.2]	557 (11.3)	159 (27.0)	398 (9.2)	**<0.001**
Flu B	36.2 [26.6, 63.5]	201 (1.6)	64 (2.3)	137 (1.4)	**<0.001**	7.1 [3.8, 12.5]	128 (2.6)	50 (8.6)	78 (1.8)	**<0.001**
RSV	73.6 [58.1, 85.7]	562 (4.4)	38 (1.4)	524 (5.3)	**<0.001**	0.6 [0.2, 1.8]	844 (16.9)	19 (3.3)	825 (18.7)	**<0.001**
RV	55.3 [39.1, 67.2]	666 (14.4)	199 (11.9)	467 (15.9)	**<0.001**	1.4 [0.4, 3.8]	909 (30.6)	73 (29.1)	836 (30.8)	0.296
AdV	41.5 [32.4, 63.1]	24 (0.5)	7 (0.4)	17 (0.6)	0.34	1.6 [0.8, 2.6]	248 (7.9)	11 (4.4)	237 (8.2)	**0.016**
hMPV	59.8 [45.0, 69.4]	90 (1.9)	18 (1.1)	72 (2.4)	**0.001**	1.2 [0.4, 2.4]	217 (6.9)	6 (7.3)	211 (2.4)	**<0.001**
PIV‐1	55.6 [42.4, 70.4]	10 (0.2)	0 (0)	10 (0.3)	**–**	2.3 [0.5, 3.4]	23 (0.8)	2 (0.8)	21 (0.8)	0.602
PIV‐2	26.0 [24.1, 34.2]	9 (0.2)	6 (0.2)	3 (0.4)	0.067	4.5 [2.9, 6.1]	17 (0.6)	3 (1.2)	14 (0.5)	0.176
PIV‐3	60.1 [47.0, 68.0]	103 (2.2)	21 (1.2)	82 (2.8)	**<0.001**	1.4 [0.6, 2.5]	162 (5.5)	15 (5.9)	147 (5.5)	0.373
PIV‐4	58.8 [41.0, 69.4]	24 (0.5)	5 (0.3)	19 (0.6)	0.077	1.8 [0.4, 2.8]	51 (1.7)	4 (1.6)	47 (1.8)	0.547

*Note*: Age is expressed in years (median [interquartile range]). Statistically significant differences (*P* < 0.01) were observed in the distribution of all studied viruses between adult and pediatric patients.

Abbreviations: AdV, adenovirus; ER, emergency room; Flu A, influenza A; Flu B, influenza B; H, hospital; hMPV, human metapneumovirus; PC, primary care; PIV, parainfluenza virus; RSV, respiratory syncytial virus; RV, rhinovirus.

## 4 DISCUSSION

In the pre‐pandemic periods, Flu and RSV followed epidemic patterns, with high prevalence during colder months. However, during the 2020–2021 season, no Flu A/B viruses were detected, which aligns with the decline in influenza circulation reported in several studies coinciding with the emergence of the novel coronavirus.^2^, ^4^, ^5^ Regarding RSV, the epidemic peak expected in the fall of 2020 was instead observed during the summer of 2021, indicating an atypical emergence of RSV beyond its usual seasonal period, which is consistent with previous research.^6^, ^7^

Among all the viruses examined, RV was the sole pathogen consistently detected throughout the entire period. Our findings are supported by several studies, some of which even demonstrate an increase in RV detection levels.^8^, ^9^, ^10^

Numerous studies have assessed the impact of non‐pharmaceutical interventions on the transmission of respiratory pathogens. Many of these studies concur on their effectiveness in reducing cases of respiratory diseases.^4^, ^5^, ^11^ However, for RV, some investigations suggest that the implemented containment measures may have a comparatively lower impact on curbing its transmission.^4^, ^5^

Spain, particularly the Community of Madrid, faced substantial consequences as a result of COVID‐19, leading to the adoption of exceptional measures for pandemic control, including a nationwide lockdown until June 2020, regional perimeter closures in the following months, and compulsory mask usage continuing until the end of 2021. Although not the primary focus of this study, a correlation can be observed between the decline in the circulation of respiratory pathogens and the non‐pharmaceutical interventions implemented.

Our study depicts the distribution of prevalent viruses among diverse populations. Although most studies focus on pediatrics,^9^, ^12^, ^13^, ^14^ our research encompasses a substantial sample of adult patients. Our findings elucidate that respiratory viruses exhibit a higher prevalence among children, with the majority of cases originating from Primary Care (Acute Respiratory Infection Surveillance Network). In contrast, positive detections among adults were predominantly observed in hospitalized or ER patients. Notably, In adults, Flu A was the most frequently detected virus, aligning with the annual surge of flu cases that overwhelm healthcare facilities during the epidemic season. Additionally, our study encompasses a large cohort of immunocompromised patients, which may explain why most positive detections of other viruses were found among hospitalized or ER patients in the adult population.

Several studies have suggested that mixed detections may lead to more severe outcomes, whereas others have not found this association.^15^ Our study identified mixed detections in 3.1% of the tested samples, with RV being the most frequently detected virus in these cases, consistent with previous studies.^13^, ^14^

Interestingly, our data showed an increase in the age of children with RSV during 2021 and 2022 compared with the pre‐pandemic period. This trend has been documented in previous studies^6^ and may be attributed to reduced exposure to RSV among younger children since the onset of the COVID‐19 pandemic.

Our study has some limitations. Not all samples were tested for all viruses. Additionally, during the beginning of the pandemic, only a limited number of samples were screened for viruses other than SARS‐CoV‐2, and a smaller number were obtained from Primary Care, potentially leading to an underestimation of certain results. However, it is worth highlighting that our findings can be generalized to the entire Community of Madrid, as we operate as a Reference Laboratory within the Epidemiological Surveillance Network. Although variations across different regions are possible, the trends closely mirror those observed in the broader context of Spain.

The COVID‐19 pandemic has profoundly impacted global health, affecting individuals across society. The evolution of the virus and implemented control measures have notably influenced the circulation of the other respiratory viruses, gradually restoring them to pre‐pandemic levels. Continuous monitoring and identification of emerging pathogens are crucial for effective infection control and prevention.

## AUTHOR CONTRIBUTIONS

**Patricia Brañas:** Conceptualization; data curation; formal analysis; methodology; writing—original draft; writing—review and editing. **Irene Muñoz‐Gallego:** Data curation; formal analysis; writing—review and editing. **Elena Espartosa:** Investigation; writing—review and editing. **Noelia Moral:** Investigation; writing—review and editing. **Guadalupe Abellán:** Investigation; writing—review and editing. **Lola Folgueira:** Conceptualization; funding acquisition; resources; supervision; writing—review and editing.

## CONFLICT OF INTEREST STATEMENT

The authors have no competing interest to declare.

### PEER REVIEW

The peer review history for this article is available at https://www.webofscience.com/api/gateway/wos/peer-review/10.1111/irv.13199.

## ETHICS APPROVAL

This study was approved by the Ethics Research Committee of our Institution (CEIm 22/468).

## Data Availability

The data that support the findings of this study are available on request from the corresponding author. The data are not publicly available because of privacy or ethical restrictions.
